# The landscape of bacterial contractile injection systems across large-scale metagenomes

**DOI:** 10.1128/spectrum.03213-24

**Published:** 2025-05-23

**Authors:** Shang Li, Jiacheng Wu, Qinghua Wang, Hongqian Cao, Lei Zhang

**Affiliations:** 1 Microbiome-X, School of Public Health, Cheeloo College of Medicine, Shandong University 162743 , Jinan, Shandong, China; 2 School of Biological Science and Technology, University of Jinan 12413, Jinan, Shandong, China; 3 Department of Physical and Chemical Inspection, School of Public Health, Cheeloo College of Medicine, Shandong University 600290 , Jinan, Shandong, China; 4 State Key Laboratory of Microbial Technology, Shandong University 520252, , Qingdao, Shandong, China; Centre National de la Recherche Scientifique, Marseille, France

**Keywords:** contractile injection systems, metagenomes, microbial interactions, ecological distribution, antibacterial activity

## Abstract

Bacterial contractile injection systems (CISs) have evolved as sophisticated mechanisms that enable bacteria to interact with and manipulate their hosts. Resembling bacteriophage tails, these systems inject effector proteins directly into target cells, facilitating complex bacterial-host interactions. Despite significant advances in understanding CISs, exploration has largely been constrained by the availability of completely sequenced microbial genomes, potentially underrepresenting their diversity in natural environments. In this study, we identified 1,129 CISs from metagenome-assembled genomes spanning 14 bacterial phyla and 1 archaeal phylum. Notably, CISs were identified in many uncultured microorganisms and were highly enriched in the phylum *Bacteroidota*. Structural gene composition varied across ecosystems, with *Bacteroidetes* injection systems (BISs) from the human digestive system lacking typical tail fiber proteins and Cis6. However, all BISs encoded homologs of *Aasi_0556*, suggesting functional similarities with T6SS^iv^. Furthermore, we identified a candidate *CIS* cargo protein, BDI_2459, containing the toxin-associated DUF4157 domain, which exhibited moderate toxin potential. We cloned and heterologously expressed BDI_2459 in *Escherichia coli*. The standalone BDI_2459 did not exhibit its activity. However, there was activity when fused with a periplasmic translocation tag.

**IMPORTANCE:**

Overall, this study expands our understanding of the ecological diversity, evolutionary adaptations, and functional roles of contractile injection systems (CISs) in microbial communities. The findings particularly highlight their adaptations to human-associated microbiomes. In addition, we conducted preliminary functional studies targeting the cargo protein BDI_2459 in *CIS* from *Parabacteroides distasonis* (CIS^Pd^). These results provide new insights into CIS-mediated bacterial interactions and pave the way for future microbiome engineering and antibacterial strategies.

## INTRODUCTION

Bacteria face numerous threats and challenges as they colonize and grow in diverse ecosystems. To adapt and thrive, many bacteria have evolved contractile injection systems (CISs)—sophisticated molecular machines that mediate interactions with their environment and host organisms (1). Resembling bacteriophage tails, CISs function as macromolecular injection devices, comprising a baseplate, a contractile sheath, and an inner tube. Upon sheath contraction, the inner tube is propelled forward, enabling the injection of effector proteins into the extracellular space or directly into the target cells, thereby facilitating complex microbial interactions with other cells and organisms (2).

CISs can be broadly classified into two major categories based on their mode of action: intracellular type VI secretion systems (T6SSs) and extracellular contractile injection systems (eCISs) (2). T6SSs are widespread among gram-negative bacteria, where they mediate contact-dependent delivery of toxic effector proteins to target cells, often as a means of competition with other microbes (3). For example, the T6SS in *Pseudomonas aeruginosa* plays a key role in bacterial competition and host-cell interactions through effector protein translocation (4). In contrast, eCISs rely on the lysis of the producer cell for release and do not require direct intercellular contact. Examples of typical eCISs include anti-feeding prophages (Afps) from *Serratia entomophila* (5), Photorhabdus virulence cassettes (PVCs) from *Photorhabdus asymbiotica* (6), and metamorphosis-associated contractile structures (MACs) from *Pseudoalteromonas luteoviolacea* (7).

Recent investigations have unveiled an array of atypical CISs characterized by their unique structural and functional attributes. An illustrative instance is the T6SS^iv^ from *Amoebophilus asiaticus*, which shares structural similarities with eCISs but functions more like a traditional T6SS, facilitating interactions with host membranes and potentially aiding in phagosome escape (8). Another novel *CIS*, termed AlgoCIS from *Algoriphagus machipongonensis*, features a specialized tail fiber, distinguishing it from typical eCIS like Afps, PVCs, and MACs (9). Additionally, *Anabaena*, a multicellular cyanobacterium, employs a distinct mechanism through thylakoid-associated CISs (tCISs), which aggregate in the cytoplasm and attach to thylakoid membranes. Under environmental stresses such as UV irradiation or high salinity, tCISs induce cell lysis, resulting in the formation of “ghost cells” (10). Furthermore, CISs have also been identified in gram-positive bacteria, such as the CIS^Sc^ system in *Streptomyces coelicolor*, which mediates cell death in response to external stress (11, 12).

The growing catalog of atypical CISs reveals the remarkable diversity of these systems in terms of structures, function, and biological roles. Genomic analyses have further highlighted this diversity. For example, a study of 11,699 bacterial genomes identified 631 putative eCIS loci, which were classified into two phylogenetic lineages (I and II) and further subdivided into six subtypes, showing widespread conservation across diverse microbial phyla, including both gram-negative and gram-positive bacteria, as well as archaea (13). Another study identified 1,425 loci across 1,249 prokaryotic genomes, underscoring the abundance of eCISs in environmental microbes. The identification of toxin-associated proteins in eCISs has further emphasized their importance in microbial community dynamics (14). Additionally, the *Bacteroidetes* injection systems (BISs), a unique *CIS* found in the human gut microbiome (HGM), have been linked to a healthy microbiome in Western populations. Research suggests that BIS genes are enriched in individuals without inflammatory bowel disease, implicating BISs in maintaining gut health (15).

Despite these advances, much remains to be understood about CISs, especially given the discovery of atypical variants that suggest considerable diversity within these systems. Current research is often limited by the availability of sequenced microbial genomes, which may introduce sampling biases and fail to capture the full diversity of CISs present in natural environments. To address this gap, our study leverages large-scale metagenomic analysis to explore the distribution, diversity, and functional roles of CISs across various microbial communities. This approach aims to provide deeper insights into how CISs mediate bacterial interactions and their ecological significance in different environments.

## MATERIALS AND METHODS

### Identification of *CIS* cluster

The initial data for identifying the *CIS* cluster were sourced from 52,515 metagenome-assembled genomes (MAGs) in the Genomes from Earth’s Microbiomes (GEM) catalog (16), 34,815 MAGs in the Ocean Microbiomics Database (OMD) (17), and 92,143 MAGs from the HGM (18). To construct the *CIS* Gene Family (CGF), we utilized eight representative CISs that have been previously reported, including PVC (GenBank accession: FM162591.1), AFP (GenBank accession: AF135182.5), AFPX (GenBank accession: KU559315.1), MAC (GenBank accession: KF724687.1), T6SS^iv^ (GenBank accession: CP001102.1), AlgoCIS (GenBank accession: CM001023.1), tCIS (GenBank accession: CP003659.1), and *Streptomyces CIS* (GenBank accession: AL645882.2).

We first performed genome annotation using Prokka (v1.11) (19), followed by gene clustering of eight *CIS* genomes using Roary (v3.13.0) (20), resulting in 84 gene families, named CIS_cluster1-84 (Data Set S1). Subsequently, we annotated the functional roles of each cluster based on previously reported *CIS* gene functions. To assess the conservation of each gene cluster, we calculated the frequency of its representative gene, which we defined as the probability of the gene appearing across all eight CISs (Data Set S1). A higher frequency indicates a higher level of conservation, suggesting a greater importance of the gene cluster. Next, we compared the CGF of each MAG individually using Diamond (v2.0.15) (21). Gene frequencies were converted into base scores, and the *CIS* score for each contig was calculated using the following formula:


$$score=Multiplier\times \left(3^{freq}-1\right).$$

Here, the multiplier represents the number of *CIS* genes identified in the contig. The contig with the highest *CIS* score was selected as the representative sequence for each MAG. A threshold was then established by considering the lowest 60% of the scores among the eight CISs. MAGs with scores above this threshold were extracted and considered as candidate CISs.

To refine our candidate *CIS* systems, we manually screened for the presence of three essential protein classes: baseplate proteins (Cis8, Cis11, and Cis12), tube proteins (Cis1, Cis5, and Cis7), and sheath initiators (Cis4 and Cis9) (22). These proteins are critical for the structural integrity and functionality of *CIS* systems. Specifically, baseplate proteins form the foundation of the *CIS* structure, tube proteins constitute the central channel, and sheath initiators bind to the initiator tubes to further stabilize the baseplate.

Based on prior comparative analyses of the protein components of CISs, T6SS^i-iii^, phages, and R-type bacteriocins (9, 10, 22, 23), we observed distinct patterns in their protein compositions. Typical T6SS^i-iii^ lack homologs of spike plug proteins (Cis6), tail fiber proteins (Cis13), and tape measure proteins (Cis14), but they contain AAA superfamily ATPase (Cis15). Conversely, phages and R-type bacteriocins lack spike plug proteins (Cis6) and AAA superfamily ATPase (Cis15), but they possess tail fiber proteins (Cis13) and tape measure proteins (Cis14). These differences are functionally significant: spike plug proteins (Cis6) stabilize the structure; tail fiber proteins (Cis13) play a role in host or target recognition; and tape measure proteins (Cis14) are crucial for determining the length of the tail assembly. Additionally, the AAA superfamily ATPase (Cis15) is associated with energy-dependent processes, such as secretion or contraction. To focus on CISs distinct from T6SS^i-iii^, phages, and R-type bacteriocins, we excluded CISs containing Cis15 but lacking Cis6, Cis13, and Cis14, as these are indicative of typical T6SS^i–iii^. Similarly, CISs containing Cis13 and Cis14 but lacking Cis15 were excluded to eliminate potential interference from phages and R-type bacteriocins. This selection strategy ensures that the refined candidate set focuses on CISs with specific protein combinations relevant to the target gene cluster family, thereby distinguishing them from related systems like T6SSs, phages, and R-type bacteriocins.

### Metagenomic mining analysis

We identified 1,129 MAGs containing *CIS* gene clusters and quantified the non-mutually exclusive hit counts for each *CIS* gene within these MAGs. These hit counts were then summed to obtain the overall count score for each *CIS* gene in each MAG. A heatmap was generated based on these count scores, with each row representing a *CIS* gene and each column representing a *CIS*. The clustering process in the heatmap is applied to the columns of the data. The columns are grouped based on similarity using Ward’s minimum variance method (Ward.D), which is designed to minimize the variance within clusters. The distance between columns is calculated using Euclidean distance. Additionally, each *CIS* was annotated with its source biome or corresponding phylum to facilitate a more intuitive visualization of the distribution patterns of *CIS* genes across different biomes and databases.

We utilized a binary presence-absence matrix to estimate the co-occurrence probabilities between gene pairs. In this matrix, each row represents a gene, each column represents a genome, and each cell contains a value of 1 if the gene is present in the genome and 0 otherwise. For each gene pair, we calculated their co-occurrence count as the number of CISs in which both genes appeared together. We then calculated the conditional probability P(j∣i) for each pair of genes *i* and *j*, defined as the ratio of the co-occurrence count of *i* and *j* to the total number of CISs in which gene *i* appeared. This approach resulted in a conditional probability matrix, where each element represents the likelihood of gene *j* being present given the presence of gene *i*. A heatmap of *CIS* gene co-occurrence was then generated from this matrix. Additionally, bar plots above the heatmap indicate the total number of CISs in which each *CIS* gene appeared.

### Phylogenetic tree analysis

The phylogenetic tree of all *CIS* proteins was constructed based on Cis7 and Cis8 protein sequences using IQ-TREE (v2.2.5) with the Q.pfam + F + I + R8 substitution model, selected on the basis of empirical state frequencies and optimized by maximum likelihood estimation. For sheath proteins, a separate phylogenetic tree was constructed using IQ-TREE with the Q.pfam + I + G4 model (24). To assess branch support and ensure robust tree topology, ultrafast bootstrapping with 1,000 replicates was conducted. The phylogenetic tree was visualized and annotated using the Interactive Tree of Life (iTOL, version 6)(25).

### Gene cluster comparison

We used the Geneviewer R package for cluster comparison and prepared gbk files, using *CIS* from *Parabacteroides distasonis* (CIS^Pd^) as the query cluster for BLASTP analysis to search for homologs in other clusters. A minimum identity threshold of 25% was used. The genes were subsequently colored based on their functional categories, and the gene names, links, and BLASTP identity values were displayed for clarity.

### Protein structure and protein complex prediction

The monomeric and trimeric structures of Aasi_0056 and BDI_2455 were predicted using ColabFold (v1.5.5)(26), a software package that provides an integrated protein structure prediction solution via a web-based interface (utilizing Google Colaboratory notebooks). Predictions were performed with the standard settings recommended by the developers, and the predicted structures were visualized in PyMOL with color coding based on calculated confidence scores (pLDDT) to indicate structural reliability (27).

### Genetic cloning and bacterial intoxication assays

The cargo (BDI_2459) gene was PCR-amplified from the genome of *Parabacteroides distasonis* ATCC8503. The PCR product was cloned into the pET28 vector using the Gibson assembly method. The cargo protein fused with the N-terminal periplasmic tag was subsequently generated based on the recombinant cargo construct (28). The periplasmic tag is prepared by overlapping PCR of three oligos (29). The plasmids were designed to express proteins without the HIS tag to prevent interference with potential toxic activity. The plasmids were then transformed into *Escherichia coli* BL21 (DE3) cells by heat shock.

To evaluate the toxic effects of the recombinant proteins, bacteria with different constructed vectors were grown overnight at 37°C in an LB medium containing kanamycin. The cultures were normalized to 0.8 OD_600_ and subsequently serially diluted. Five microliter of serial dilutions was spotted on LB agar containing related antibiotics (50 µg/mL kanamycin) and without/with an inducer (0.1 mM IPTG). Bacteria carrying the empty vector (pET28) and GFP protein were used as negative controls.

## RESULTS

### Identification of *CIS* clusters

**Fig 1:**
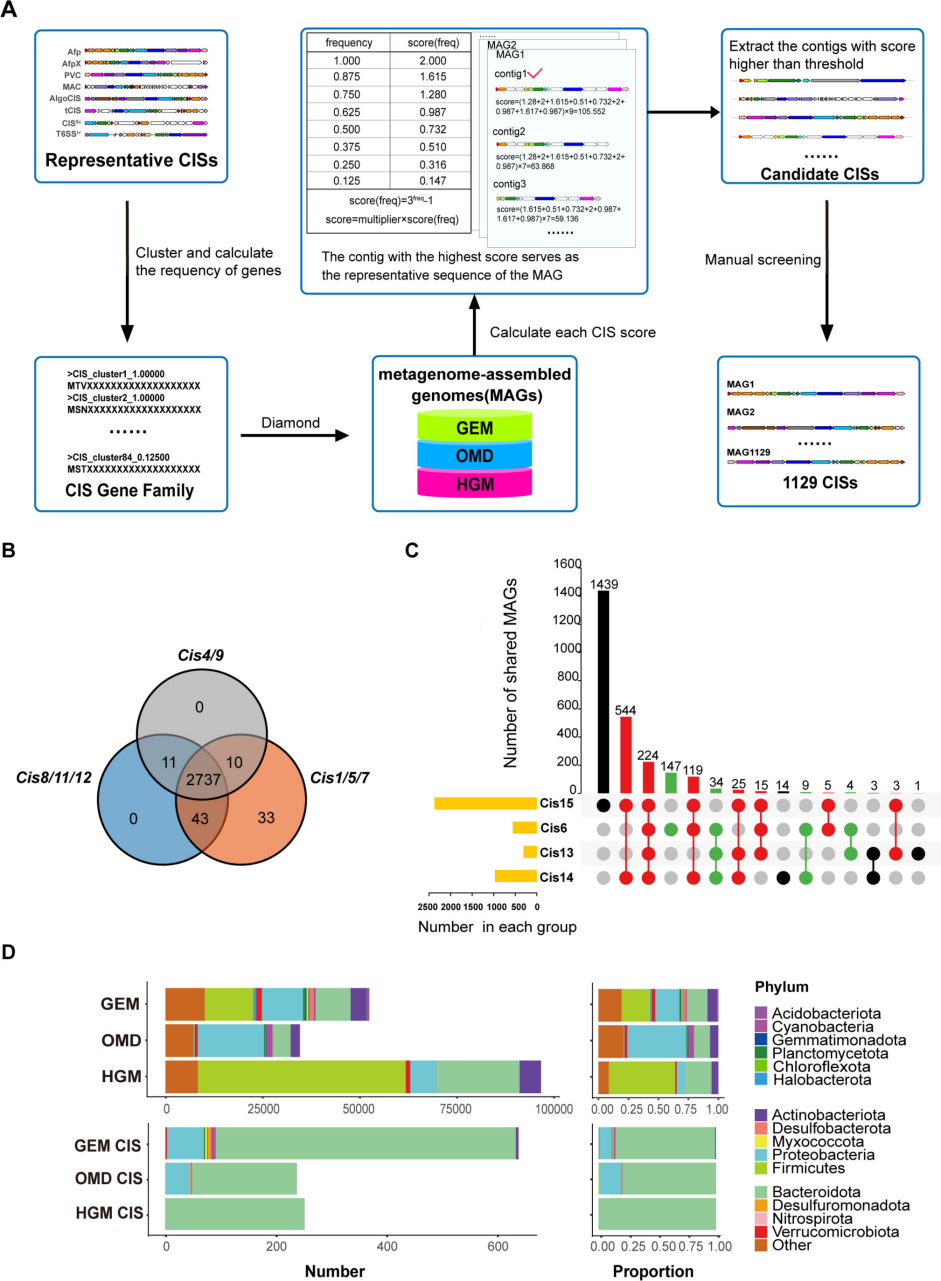
Identification of *CIS* clusters across three metagenomic databases. (**A**) Overview of the workflow used to identify *CIS* clusters within public metagenomic databases. (**B**) Results of manual filtering. The Venn diagram illustrates the preliminary screening results, which identified 2,737 MAGs. (**C**) The upset plot shows the final results after removing false positives, with 1,129 MAGs identified as containing *CIS* gene clusters. (**D**) The phylum-level species composition of the databases and the phylum-level species composition of the CISs identified from the databases.

We initially constructed CGFs using eight previously reported CISs (Fig. 1A). The *CIS* genes were clustered into 84 gene families (Data Set S1). By calculating the gene frequency, we identified 2 core genes (frequency: 0.99–1), 18 shell genes (frequency: 0.15–0.95), and 64 cloud genes (frequency: 0–0.15). Previous studies on PVC, a representative eCIS, have demonstrated that single deletions of genes encoding baseplate subunits (Pvc8, Pvc11, and Pvc12), tube proteins (Pvc1, Pvc5, and Pvc7), and sheath initiators (Pvc4 and Pvc9) prevent the production of syringe-like particles (22). Based on this, we initially screened candidate CISs and excluded those lacking any of these gene categories, resulting in 2,737 candidate CISs (Fig. 1B). To further focus on *CIS* systems distinct from T6SS^i–iii^, phages, and R-type bacteriocins, we conducted a second screening based on the comparative analysis of *CIS* components with these potential interferences as reported in previous studies (9, 10, 22, 23). These studies have shown that T6SS^i–iii^ structures contain homologs of Cis15 but lack homologs of Cis6, Cis13, and Cis14, while phages and R-type bacteriocins contain homologs of Cis13 and Cis14 but lack homologs of Cis6 and Cis15. Therefore, we used the presence of Cis15 and the absence of Cis6, Cis13, or Cis14 to exclude CISs that likely contained T6SS^i–iii^. Additionally, we removed CISs with Cis13 and Cis14 but lacking Cis15 and Cis6 to avoid potential interference from phages and R-type bacteriocins (Fig. 1C). For further reference, we have summarized the comparative analysis of reported *CIS* components (Table S1). Ultimately, we identified 1,129 CISs distributed across 14 bacterial phyla and one archaeal phylum (Data Set S3). Among these, 639 CISs were derived from the GEM catalog, 238 from the OMD, and 252 from the HGM database. In contrast to previous CISs identified from whole genomes, many of the CISs we identified originated from uncultured microorganisms (Data Set S4). Analysis of the species composition of CISs across these three distinct databases revealed that CISs were widely distributed in the phylum *Bacteroidota* (Fig. 1D). Compared to specific metagenomic databases, such as OMD and HGM, the GEM catalog, which encompasses metagenomic data from diverse habitats, uncovered a greater diversity of CIS-associated species spanning 14 bacterial phyla and one archaeal phylum. CISs identified from the ocean microbiome were enriched not only in *Bacteroidota* but also in the phylum *Proteobacteria*. In contrast, CISs from the human gut microbiome were predominantly associated with *Bacteroidota*, with only a single *CIS* observed in the Proteobacteria phylum, consistent with previous findings on the BISs observed in the human gut (15).

### Environmental and geographic distribution of CISs

**Fig 2:**
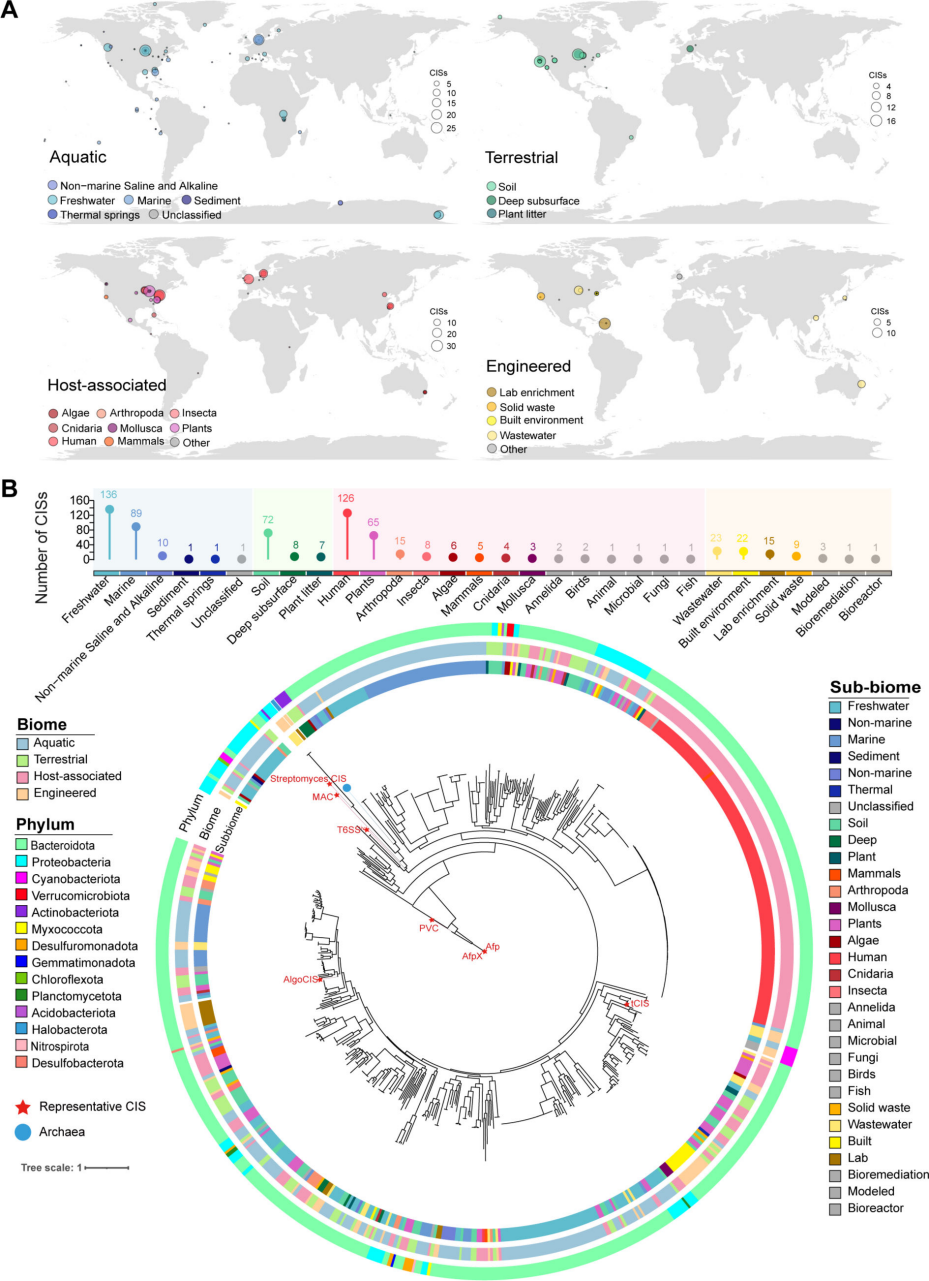
Geographic and phylogenetic distribution of CISs in the GEM. (**A**) Geographic distribution of CISs within each biome. The map was created using ggplot2. (**B**) The number of CISs within each biome and phylogenetic tree of the CISs. The protein sequences of *Cis7* and *Cis8* from each cluster were concatenated, aligned, and used to construct the phylogenetic tree. *CIS* clusters derived from archaeal genomes are highlighted with solid blue circles, whereas previously reported CISs are marked by red pentagons. The outer strips were color coded according to their respective phylum, biome, and sub-biome.

The GEM catalog, a comprehensive metagenomic data set encompassing diverse microbial habitats and geographic locations, shows some overlap with the OMD and HGM databases, which focus on specific ecosystems. To illustrate the global distribution of CISs, we mapped 639 CISs identified from the GEM data set. Based on the GEM classifications, CISs were categorized into four major groups according to microbial isolation sources, ecosystems, habitats, lifestyles, and host organisms: aquatic environments (238), host-associated environments (240), terrestrial environments (88), and engineered environments (75; Fig. 2A). This distribution highlights the widespread presence of CISs across diverse ecosystems. Compared to the entire GEM data set, the five sub-biomes with the highest relative abundance of CISs were plant litter (8.86%), plants (5.75%), soil (2.71%), sediment (2.17%), and solid waste (2.06%). In contrast, only 0.77% of human-associated MAGs contained *CIS*, all of which were detected in the digestive system (Fig. 2B; Data Set S5).

To delineate evolutionary relationships among CISs, we constructed a phylogenetic tree using concatenated Cis7-Cis8 protein sequences. This dual-gene approach was selected because (i) Cis8 showed universal presence across all 639 analyzed *CIS* clusters (Source Data Fig. S3C), ensuring comprehensive representation; (ii) Cis7 co-occurred in 638 clusters, providing complementary phylogenetic signals. The resulting phylogeny confirmed the established division of CISs into subtypes I and II (13) (Fig. S1A). Interestingly, the majority of the reported *CIS* resided on narrow branches of the phylogenetic tree (Fig. 2B), hinting at a potentially greater diversity of CISs than previously acknowledged. Moreover, CISs derived from human-associated sources formed distinct clusters on the phylogenetic tree, with their habitats exclusively linked to the digestive system. Conversely, CISs derived from marine and terrestrial environments exhibited a more dispersed distribution, mirroring the vast microbial diversity and the variability inherent in their habitats. These findings emphasize the ecological and evolutionary differentiation of CISs across different environments and host-associated systems.

### Diverse distribution patterns of *CIS* genes across ecosystems

**Fig 3:**
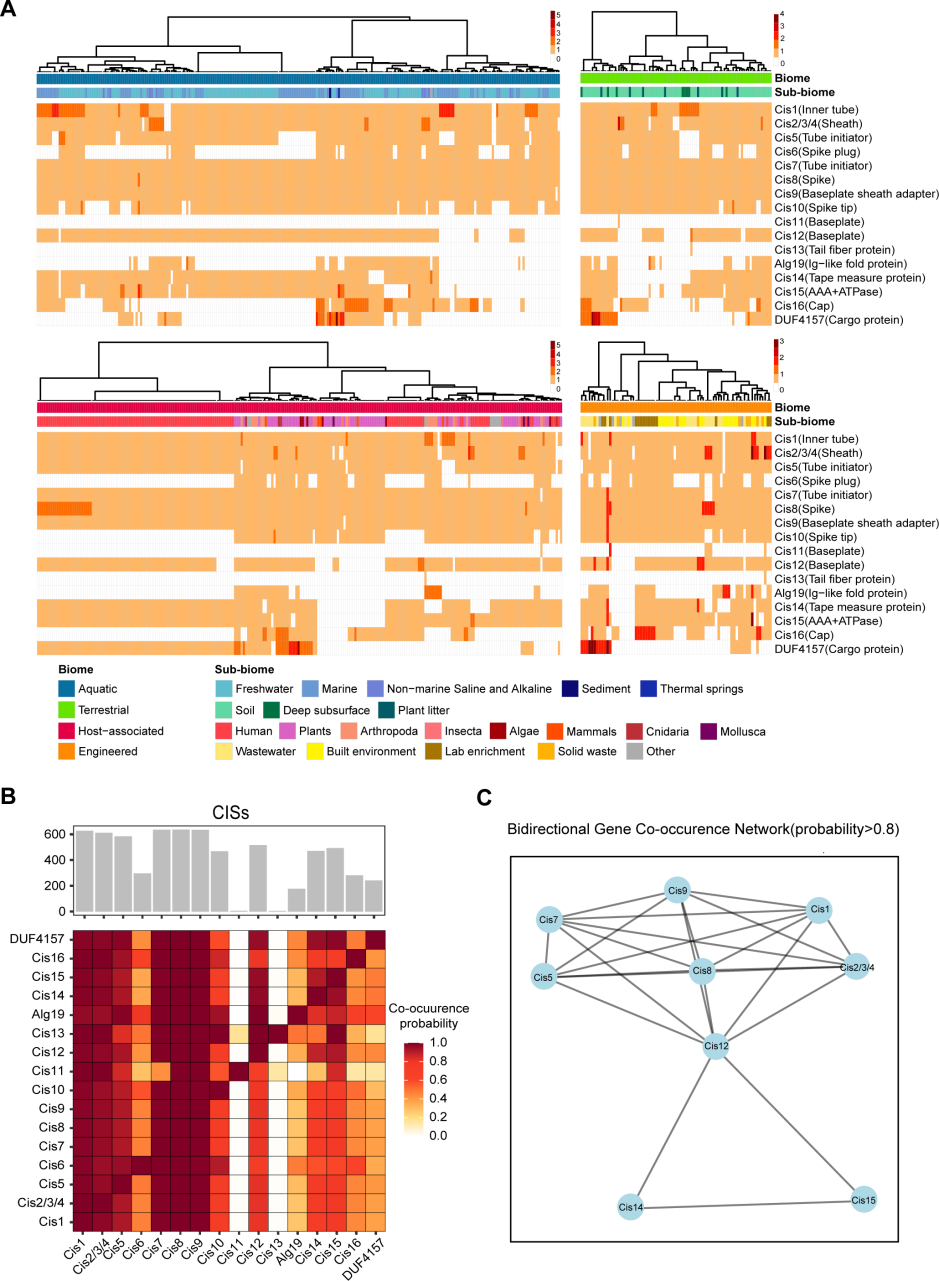
Coverage and co-occurrence analysis of CISs in the GEM. (**A**) Coverage map of *CIS* genes in *CIS* gene clusters from the GEM. (**B**) Co-occurrence of *CIS* genes from GEM. Each cell in the heatmap, a*_ij_*, represents the probability that *CIS* gene *j* is encoded in a *CIS* given that *CIS* gene *i* is encoded. The bar plots at the top and right of the heatmap show the number of CISs in which each *CIS* gene is detected. (**C**) Gene network map with bidirectional co-occurrence probability greater than 0.8.

To gain further insight into the prevalence and distribution of *CIS* genes across diverse ecosystems, we employed the GEM database to examine the abundance of *CIS* genes in aquatic, host-associated, terrestrial, and engineered environments. A coverage map of *CIS* genes was constructed (Fig. 3A), which revealed that all *CIS* clusters contain at least four structural *CIS* genes. It is noteworthy that Cis1, Cis7, Cis8, and Cis9 are present in 98.6% of these clusters (Fig. 3B). From the *CIS* gene coverage map, it was evident that there were notable differences in the distribution and co-occurrence patterns of *CIS* genes across distinct ecosystems. *CIS* genes in aquatic environments share a similar distribution pattern with those in OMD, collectively highlighting the characteristics of *CIS* distribution in marine microbial communities. Specifically, Cis13 tail fiber protein is enriched in marine-derived *CIS*, displaying a higher correlation with cis11 (Fig. S2A through C). In contrast, CISs from terrestrial and engineered environments exhibit high diversity and widespread distribution. Notably, CISs derived from the digestive system lack Cis6, Cis13, and AIg19. This conclusion is based on our systematic analysis of eight previously reported *CIS* gene clusters. Using Roary for gene clustering, we identified 84 gene families (CIS_cluster1-84; Data Set S1). Among these, CIS_cluster40 (Pvc6) and CIS_cluster80 (AIg6), encoding spike plug proteins, were annotated as Cis6. CIS_cluster14 (Afp13, Pvc13, and AfpX13), encoding tail fiber proteins, was annotated as Cis13, while CIS_cluster84 (AIg19), which exhibits tail fiber functionality in the AIgCIS, was annotated as AIg19. In *CIS* gene clusters from the digestive system, we detected no homologous sequences to these reference gene families, leading us to conclude the absence of these key genes. This unique gene depletion pattern is also observed in HGM-associated CISs. The gene co-occurrence heatmaps and networks in HGM CISs display a more centralized and tightly interconnected architecture (Fig. S2D through F), distinct from other biomes, potentially reflecting adaptations to the specialized gut environment. In addition, we observed that DUF4157, a potentially existing cargo protein, shows a higher correlation with AIg19 than with Cis13 (Fig. 3B). The bidirectional gene co-occurrence network highlights key genes within *CIS* gene clusters from GEM (Fig. 3C). These results not only elucidate the distribution patterns in various ecosystems but also provide crucial insights into their potential functional adaptations and ecological and evolutionary significance.

### The role of tail fiber proteins in evolution and specialization

**Fig 4:**
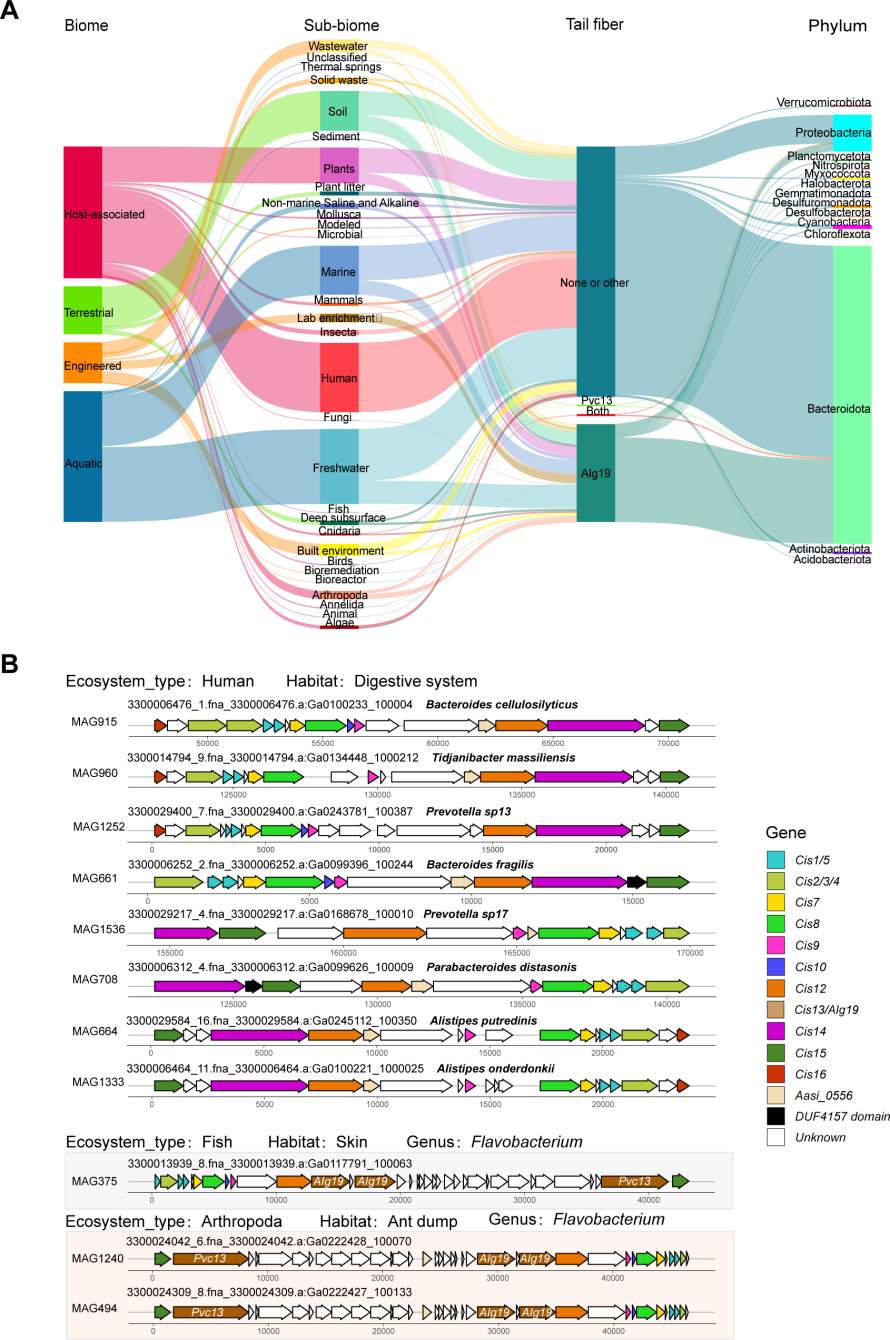
Association of *CIS* tail fiber proteins with different bacterial phyla and microbial communities. (**A**) Sankey plot showing the association of the tail fiber gene with different biomes, showing its distribution across different environments and classification at the phylum level. (**B**) *CIS* gene clusters derived from the human digestive system and those containing both *the Pvc13* and *AIg19* homologous genes.

Ecosystems profoundly influence the structural evolution of CISs, with tail fiber proteins playing a critical role in target recognition within classical eCISs. Consequently, the study of tail fiber proteins is of great importance for the discovery of novel CISs. In our earlier work on constructing *CIS* gene cluster families, we classified tail fiber proteins into two categories based on current reports: Cis13 (CIS_cluster14, homologous to Afp13, Pvc13, and AfpX13) and AIg19 (CIS_cluster84). By associating the tail fibers of identified CISs with different biological communities and bacterial phyla, notable patterns were observed. Specifically, CIS-producing bacteria in the human digestive system belonged to the phylum *Bacteroidota* (Fig. 4A). However, these CISs lacked both Cis13 and AIg19 tail fiber proteins. Instead, these BISs uniformly contained a gene homologous to that of Assi_0556 (Fig. 4B). Furthermore, a unique *CIS* produced by the *Flavobacterium* genus, found in the epidermal mucus of fish skin, exhibited tail fibers resembling both Pvc13 and AIg19. Intriguingly, this unique *CIS* was also detected in the microbial communities of ant dumps within Arthropoda (Fig. 4B). These findings reveal the ecological and functional diversity of CISs, highlighting the role of tail fiber proteins in shaping their specialization and evolutionary trajectories across distinct environments.

### BISs from the human digestive system are structurally similar to T6SS^iv^

**Fig 5:**
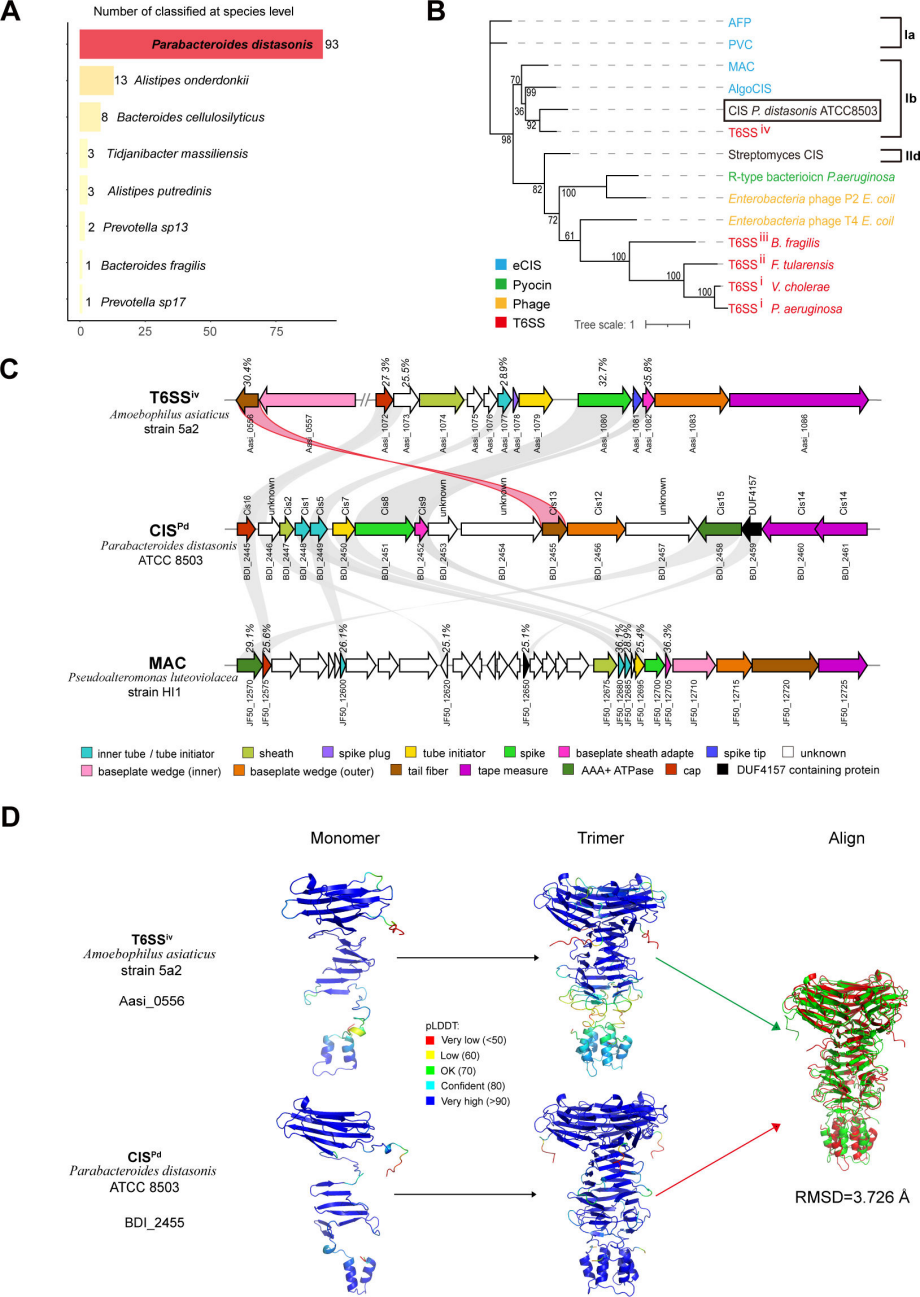
Homology and structural analysis of tail fibers in BIS and T6SS^iv^. (**A**) The number of species classified at the species level within BIS. (**B**) Phylogenetic tree based on phylogenetic analysis of putative sheath proteins. Subclades Ia, Ib, and IId are based on the dbeCIS database. (**C**) Cluster comparisons of BIS, T6SS^iv^, and MAC were performed, with CIS^Pd^ serving as a representative BIS. (**D**) Monomeric and trimer protein structures of the T6SS^iv^ tail fiber (Aasi_0056) and its homolog in CIS^Pd^ (BDI_2455) predicted by ColabFold (26).

We analyzed the top 10 species classified at the species level within the BISs from the human digestive system and found that *Parabacteroides distasonis* ranked the highest with a count of 93 (Fig. 5A). Using the same identification method, we identified a *CIS* gene cluster in the genome of the standard strain *Parabacteroides distasonis* ATCC8503. As a representative of BIS, the CIS^Pd^ was selected for phylogenetic analysis of its sheath protein in comparison with those of other *CIS* types and bacteriophages (Fig. 5B). The results demonstrated that the sheath protein of CIS^Pd^ is distinct from the T6SS of subtypes 1, 2, and 3, as well as R-type bacteriocins and bacteriophages. Instead, CIS^Pd^ forms a monophyletic group with T6SS^iv^ and MAC and is classified into group Ib.

For further investigation, we selected CIS^Pd^ as the query cluster and performed BlastP analysis to identify homologs in T6SS^iv^ and MAC (Fig. 5C). In the resulting schematic, gray lines indicate homologous genes with ≥25% identity. Genes with the same function are filled with the same color, while those with unknown functions are shown in white. Notably, the BDI_2455 protein from CIS^Pd^ shares homology with the Aasi_0556 protein in T6SS^iv^ (highlighted in red), with a 30.4% identity. This observation is consistent with our previous finding that homologs of Aasi_0556 are present within the BIS gene cluster. The gene encoding the Aasi_0556 protein in T6SS^iv^, referred to as Cis13, serves a role analogous to the tail fiber gene, potentially aiding in anchoring T6SS^iv^ to the inner membrane. To further explore this, we conducted a multiple sequence alignment of genes homologous to Aasi_0556 in the previously identified BIS gene clusters (Fig. S3A). Additionally, we predicted the three-dimensional structures of Aasi_0556 and BDI_2455 (Fig. 5D). The results indicate that the two proteins share 30.4% sequence similarity, with a monomer root-mean-square deviation (RMSD) of 4.488 Å (Fig. S3B). However, the RMSD decreases to 3.726 Å in the trimer configuration. This suggests that, despite their low sequence homology, these two proteins may exhibit functionally relevant conservation at higher-order structural levels, such as in their multimeric forms, thus supporting the hypothesis of a shared functional mechanism between them.

### Functional exploration of CIS^Pd^ cargo protein

**Fig 6:**
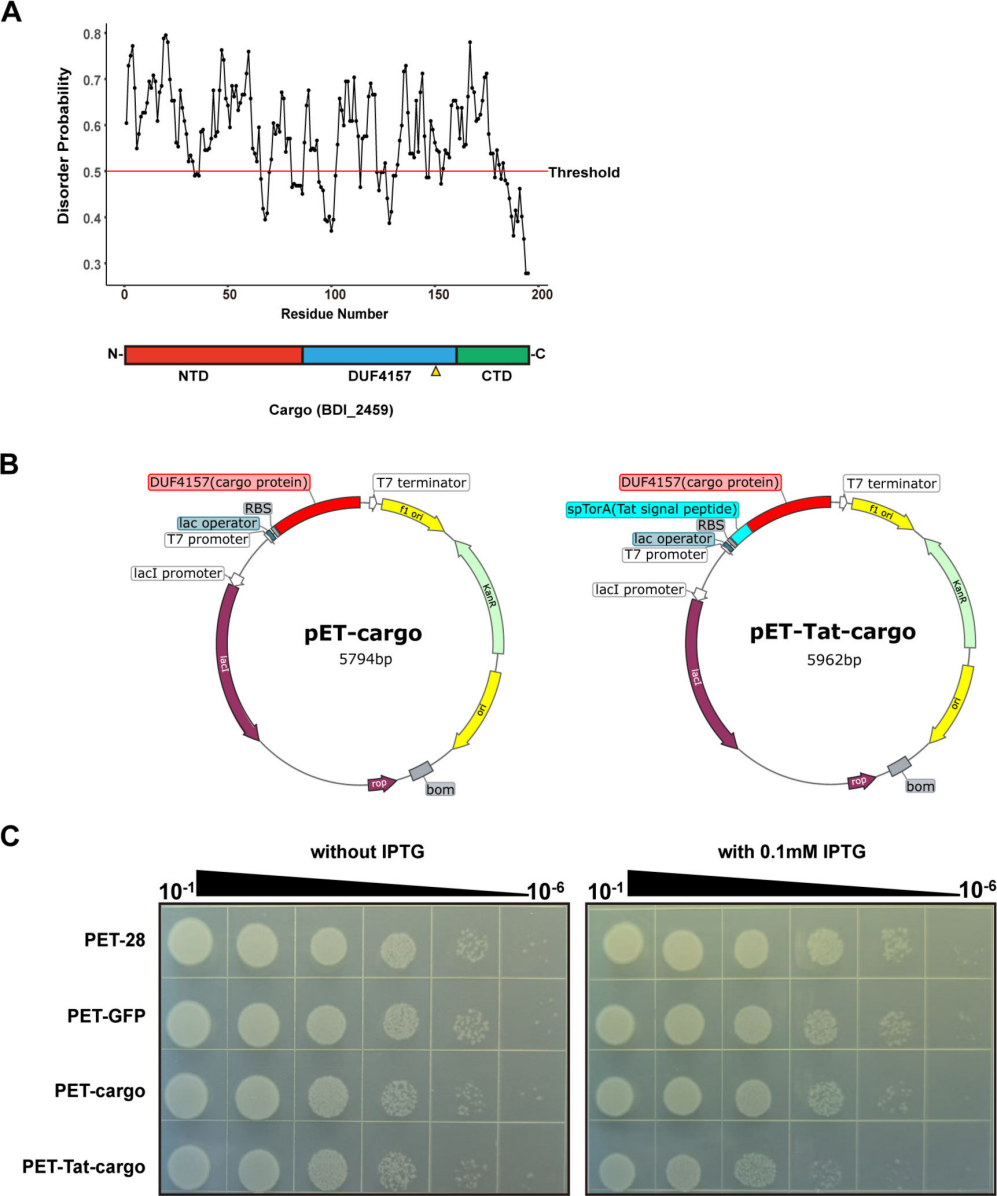
Identification and characterization of CIS^Pd^ cargo protein. (**A**) Schematic and disorder probability plot showing the domain organizations and disorder properties of putative cargo protein (BDI_2459). The positions of the putative metalloprotease motif (HExxH) are highlighted by yellow triangles. (**B**) Schematic representation of the constructed plasmids. (**C**) Bacterial spot assays show that the expression of the full-length cargo had no effect on *E. coli* (the columns represent the serial dilutions from 10^−1^ to 10^−6^ with a dilution factor of 10 per step). However, the fusion of the cargo with the periplasmic tag showed a certain level of killing effect when induced with 0.1 mM IPTG. The pET28a and pET-GFP vectors serve as negative controls. This experiment was performed three independent times with representative images shown.

In addition to structural genes, the cargo proteins carried by *CIS* have become a major focus of research. The DUF4157 domain has previously been identified as a characteristic domain of toxin proteins in eCIS and T6SSs (30). In the CIS^Pd^ gene cluster, we identified a protein of unknown function, BDI_2459, which contains the DUF4157 domain. We designated this protein as Cargo. To further investigate its potential role as a toxin, we employed a previously reported machine learning model designed to identify eCIS-related toxins (31). The model predicted Cargo to be a toxin protein, although the prediction score was relatively low at 0.81. Its function was hypothesized to resemble that of a DNA segregation ATPase, similar to FtsK/SpoIIIE-like proteins (Data Set S6). We conducted an analysis of the domain organization and disorder properties of Cargo (BDI_2459) using a disorder probability plot (Fig. 6A). Interestingly, Cargo from CIS^Pd^ did not exhibit the typical disordered N-terminal domain (NTD) characteristic of previously characterized toxin proteins. To assess its potential toxin activity, we recombinantly expressed Cargo in *E. coli* (Fig. 6B). The results showed that the standalone Cargo protein did not exhibit any antibacterial activity. However, it showed weak activity when fused with a periplasmic translocation tag (Tat system; Fig. 6C).

## DISCUSSION

This study provides an in-depth investigation of CISs, examining their distribution, structural diversity, and functional adaptations across various ecosystems. Our findings offer new insights into the ecological and evolutionary roles of CISs, with particular emphasis on their presence in the human digestive system. Metagenomic technologies have greatly enhanced our ability to explore complex microbial communities, surpassing the limitations of traditional culturing methods (32, 33). We identified 1,129 CISs across different ecosystems through large-scale metagenomic analysis, many of which are derived from uncultivated microorganisms. Our results suggest that CISs are broadly distributed within the phylum *Bacteroidota*, with notable enrichment in Proteobacteria in marine environments, indicating distinct phylogenetic preferences linked to specific ecosystems. The global distribution of CISs across aquatic, host-associated, terrestrial, and engineered environments underscores their ecological versatility. However, variations in structural gene composition, such as the absence of Cis6 and tail fiber protein genes in human-associated CISs, suggest adaptations to specific ecological niches. This highlights how ecological environments shape the distribution patterns of *CIS* genes.

The structural diversity of tail fiber proteins among CISs underscores their functional specialization in mediating host-microbe interactions. For instance, the unique CISs detected in fish skin microbiomes and ant dump communities highlight the role of tail fiber proteins in mediating interactions with specific hosts or microbial communities. Notably, BISs from the human digestive system lack typical tail fiber proteins, such as Cis13 and AIg19, and instead encode homologs of Aasi_0556. This implies that BISs may not be released into the external environment to recognize target cells through tail fibers, as seen in eCISs. The homology between Aasi_0556 in T6SS^iv^ and BDI_2455 in CIS^Pd^, along with their similar three-dimensional structures, suggests that they may share a common functional mechanism. It has been reported that T6SS^iv^ is anchored to the membrane via a unique inner membrane anchoring complex (2, 8). Whether BIS and the T6SS^iv^ system play analogous roles in cargo protein delivery remains to be investigated further through experimental research.

The role of cargo proteins in CISs has emerged as a critical area of research, as *CIS* gene clusters can serve as a treasure trove for discovering antibacterial toxins (1). Through the exploration of CISs, we have identified a large number of proteins containing the DUF4157 domain, typically associated with toxins (14). Notably, we identified Cargo (BDI_2459), a protein containing this toxin-associated domain within CIS^Pd^. Previously characterized toxin proteins typically exhibit a disordered NTD, but this feature is absent in BDI_2459. The disordered N-terminal domain in eCIS functions as a signal peptide, recognized by Pvc15 homologs and subsequently loaded into the eCIS particle (34). This signal peptide has been demonstrated to be essential for the loading and translocation of toxin proteins within eCIS (31, 35). Our experimental investigations revealed that the standalone Cargo protein lacked antibacterial activity in *E. coli*. However, it was only when fused with a translocation tag that its activity became apparent upon overexpression. We speculate that Cargo may be a protein exerting bactericidal effects in the periplasmic space. However, this hypothesis cannot be confirmed solely by the experiment of heterologous overexpression of the protein in *E. coli*, and we acknowledge the limitations of this approach. The growth inhibition we observed could also be partially attributed to metabolic burden or the formation of insoluble aggregates. It is also possible that the proteins themselves are not toxic. In fact, machine learning models designed to predict eCIS-related toxins suggested that Cargo functions as a DNA segregation ATPase, similar to FtsK/SpoIIIE proteins (31). In natural biological systems, FtsK/SpoIIIE-like proteins facilitate DNA segregation and translocation and are inherently non-toxic (36). Nevertheless, mislocalization or overproduction of FtsK/SpoIIIE-like proteins can disrupt vital cellular processes, leading to growth defects. This might be the reason why Cargo exhibits a slight antibacterial effect when overexpressed in the periplasm. Further research is needed to better understand the function of this protein.

In conclusion, this study provides a comprehensive analysis of CISs, emphasizing their diversity, ecological distribution, and functional adaptations. BIS, a unique type of *CIS* derived from human gut microbes, is closely associated with T6SS^iv^, which targets eukaryotic cells, and the current study suggests that BIS prefers to target human cells. In addition, we have conducted a preliminary exploration of the function of the BDI_2459 protein within CIS^Pd^. Further studies are necessary in the future to elucidate the mechanism of action of this protein. In the meantime, identifying target entities for BIS will aid in its engineering application of microbiome engineering and antimicrobial strategies.

## Data Availability

The data sets supporting the conclusions of this article are available in the Genomes from Earth’s Microbiomes (GEM) catalog (https://portal.nersc.gov/GEM), the Ocean Microbiomics Database (OMD; https://microbiomics.io/ocean/), and the Human Gut Microbiota (HGM; ftp://ftp.ebi.ac.uk/pub/databases/metagenomics/umgs_analyses).

## SUPPLEMENTAL MATERIAL

Supplemental tables and figures spectrum.03213-24-s0001.pdf Tables S1 and S2; Fig. S1 to S3.

Supplemental source data spectrum.03213-24-s0002.xlsx Source data for figures.

Supplemental data sets spectrum.03213-24-s0003.xlsx Data Sets S1 to S6.

ASM does not own the copyrights to Supplemental Material that may be linked to, or accessed through, an article. The authors have granted ASM a non-exclusive, world-wide license to publish the Supplemental Material files. Please contact the corresponding author directly for reuse.
