# Supplementary material for: The landscape of bacterial contractile injection systems across large-scale metagenomes
Source: Microbiol Spectr. 2025 May 23;13(7):e03213-24. doi: 10.1128/spectrum.03213-24 (PMC12210884; doi:10.1128/spectrum.03213-24)
Supplement: Supplemental tables and figures — Tables S1 and S2; Fig. S1 to S3. [file spectrum.03213-24-s0001.pdf]

The Landscape of Bacterial Contractile Injection Systems across Large-Scale Metagenomes

Supplementary Information

Supplementary tables 1-2

Supplementary figures 1-3

Supplementary Table 1 Comparison of the CIS components in representative CISs

S. entomophila	*S. proteamaculans*	*Photorhabdus*	*P. luteoviolacea*	*Anabaena*	*A. machipongonensis*	*S. coelicolor*	T6SS^{IV}	T6SS	T4 phage	R-type pyocin	Function
Afp1	AfpX1	Pvc1	AHX39701.1	all3324	Alg1	SCO4248	Aasi_1077	TssD	gp19	PA0623	inner tube
Afp2	AfpX2	Pvc2	AHX39703.1	all3325	Alg2	SCO4253	Aasi_1074	TssB/C	gp18	PA0622	sheath
Afp3	AfpX3	Pvc3						TssB/C	gp18		sheath
Afp4	AfpX4	Pvc4						TssB/C	gp18		sheath
Afp5	AfpX5	Pvc5	AHX39702.1	all3323	Alg5	SCO4252			gp54		tube initiator
Afp6	AfpX6	Pvc6		all3322	Alg6		Aasi_1078				spike plug
Afp7	AfpX7	Pvc7	AHX39698.1	all3321	Alg7	SCO4247	Aasi_1079		gp48 (gp53-LysM)	PA00626, PA0627 (LysM)	tube initiator
Afp8	AfpX8	Pvc8	AHX39697.1	all3320	Alg8	SCO4246	Aasi_1080	VgrG	gp27/5	PA0628/0616	spike
Afp9	AfpX9	Pvc9	AHX39696.1	all3318	Alg9	SCO4245	Aasi_1082	TssE	gp25	PA0617	baseplate sheath adapter
Afp10	AfpX10	Pvc10		all3319	Alg10		Aasi_1081	PAAR	gp5.4	PA0616	spike tip
Afp11	AfpX11	Pvc11	AHX39695.1	all3317	Alg11	SCO4244	Aasi_0557	TssF	gp6/7	PA0618	baseplate wedge (inner)
Afp12	AfpX12	Pvc12	AHX39694.1	all3315	Alg12		Aasi_1083	TssF/G	gp6	PA0619	baseplate wedge (outer)
Afp13	AfpX13	Pvc13	AHX39693.1/ AHX39723.1	all3316	Alg19		Aasi_0556		gp9/10/11/12	PA0620	tail fiber
Afp14	AfpX14	Pvc14	AHX39690.1	all3313	Alg14		Aasi_1806		gp29	PA0625	tape measure
Afp15	AfpX15	Pvc15	AHX39725.1		Alg15	SCO4259		ClpV			AAA+ ATPase
Afp16	AfpX16	Pvc16	AHX39724.1	all3327/3326	Alg16A/B	SCO4260	Aasi_1072	TssA	gp15	PA0615	cap

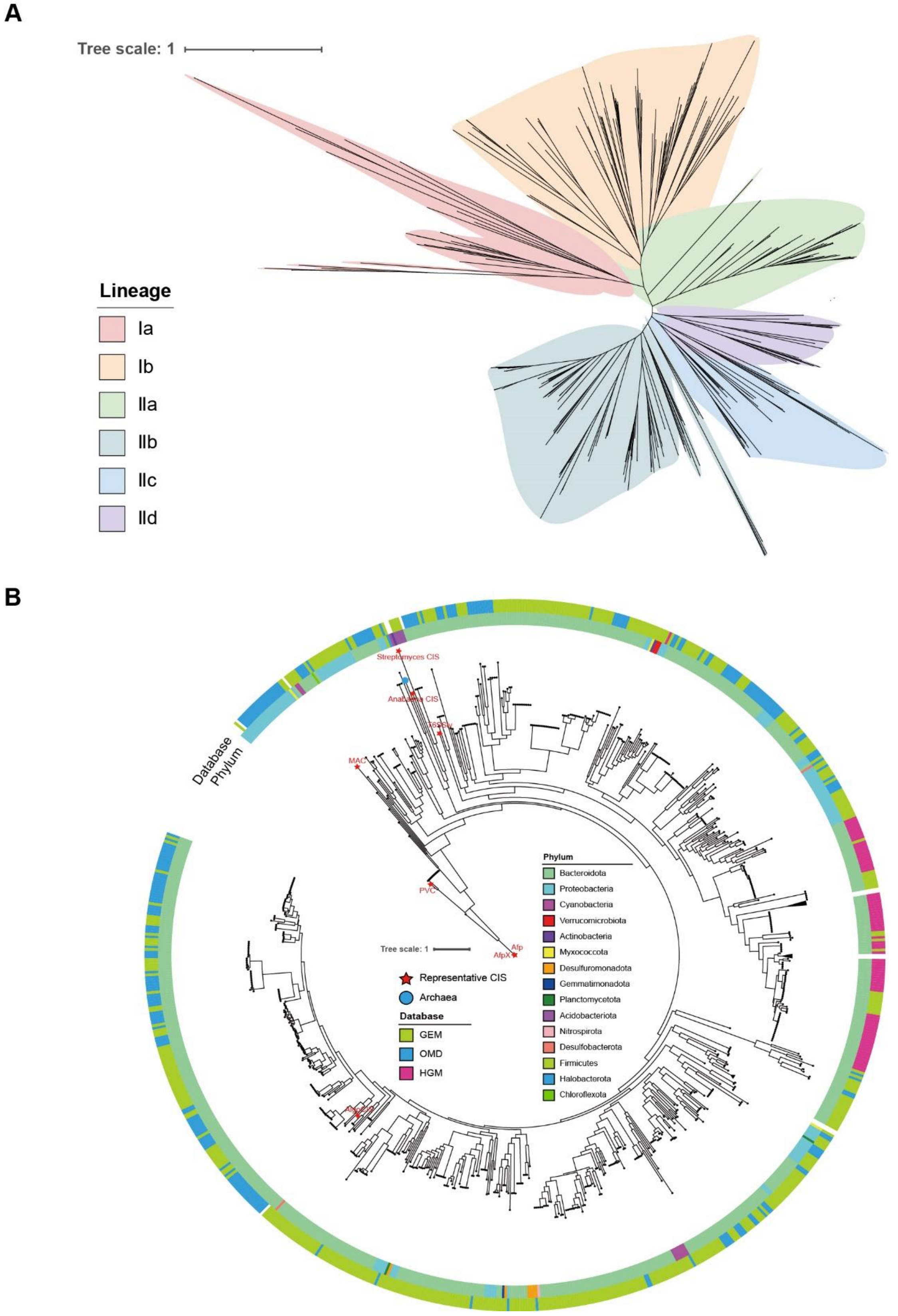

Supplementary fig.1 Phylogenetic tree of CISs. A The unrooted phylogenetic tree of CISs in the GEM database. Different colored blocks represent different lineages. **B** Phylogenetic tree of CISs from three databases, constructed using the Cis8 sequence.

CIS clusters derived from archaeal genomes are highlighted with solid blue circles, whereas previously reported CISs are marked by red pentagons. The outer strips were color-coded according to their respective phylum and database.

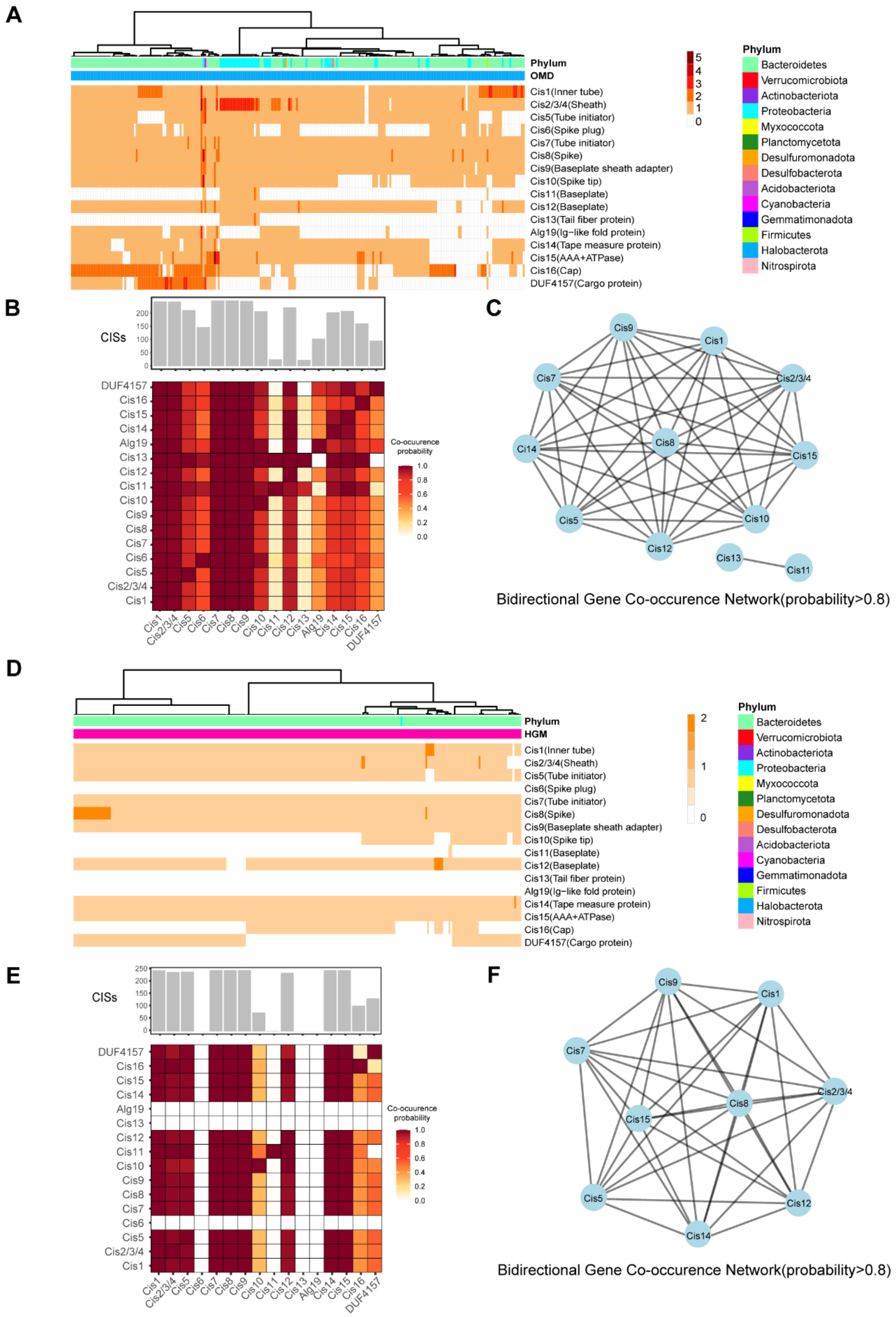

Supplementary fig.2 The abundance of CIS genes in OMD and HGM. A Coverage map of CIS genes in CIS gene clusters from the OMD. **B** Co-occurrence of CIS genes from OMD. Each cell in the heatmap, a_{ij}, represents the probability that CIS gene j is encoded in a CIS given that CIS gene i is encoded. The bar plots at the top and right of

the heatmap show the number of MAGs in which each CIS gene is detected. **C** Gene network map with bidirectional co-occurrence probability greater than 0.8 in OMD. D Coverage map of CIS genes in CIS gene clusters from the HGM. **E** Co-occurrence of CIS genes from HGM. Each cell in the heatmap. **F** Gene network map with bidirectional co-occurrence probability greater than 0.8 in HGM.

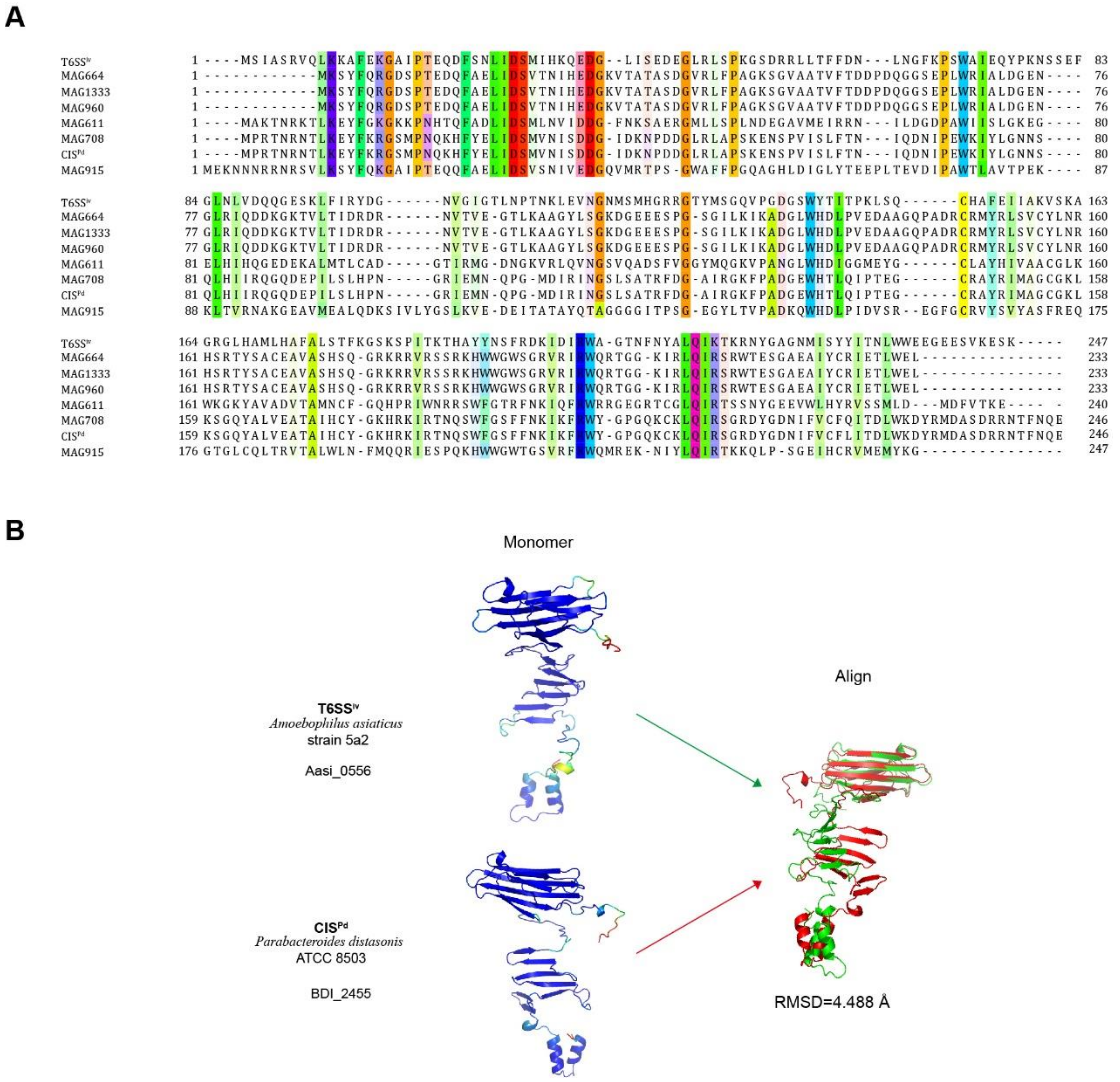

Supplementary fig.3 The tail fiber of BIS and T6SS^{iv} are homologous. A Multiple sequence alignment of tail fiber proteins from Bacteroidetes injection systems (BISs) and T6SSiv, the conservation threshold was adjusted to 60%. **B** Monomer structures of T6SSiv tail fiber (Aasi_0056) and its homolog in CISPd (BDI_2455) predicted by ColabFold.

Supplementary Table 2 List of primers

Primer	Sequence
**F-pET28**	5'- GATCCGGCTGCTAACAAAGC -3'
**R-pET28**	5'- GGTATATCTCCTTCTTAAAGTTAAACAAAAT -3'
**F-pET-Cargo**	5'- ctttaagaaggagatataccATGAATGCGGATCGGATACAGA -3'
**R-pET-Cargo**	5'- cagtggtggtggtggtggtgTCATAAGACAGAAGCAGCTTTAGCG -3'
**F-Tat-1**	5'- atggcgaacaataacgatctctttcaggcatcacgtcggcgttttctggcacaac -3'
**F-Tat-2**	5'- gttttctggcacaactcggcggcttaaccgtcgccgggatgctggggccgtcatt -3'
**R-Tat**	5'- acgcgccgcttgcgccgcagtcgcacgtcgcggcgttaacaatgacggccccagc -3'
**F-pET-Tat**	5'- ctttaagaaggagatataccATGGCGAACAATAACGATCTCTT -3'
**R-Cargo-Tat**	5'- tgtatccgatccgcattACGCGCCGCTTGCGC -3'
**F-Tat-Cargo**	5'- cgtAATGCGGATCGGATACAGATTC -3'
